# The Effects of Treatment with Icariin on Immune Tolerance in the Recurrent Spontaneous Abortion Mice

**DOI:** 10.1007/s43032-023-01185-0

**Published:** 2023-03-08

**Authors:** Fang Peng, Zhongyu Han, Haoran Chen, Qinxiu Zhang, Chi Liu, Xin Liang

**Affiliations:** 1 Chengdu University of Traditional Chinese Medicine, Chengdu, China; 2 Department of Nephrology, Sichuan Academy of Medical Science and Sichuan Provincial People’s Hospital, Sichuan Renal Disease Clinical Research Center, University of Electronic Science and Technology of China, Chengdu, China; 3 Chinese Academy of Sciences Sichuan Translational Medicine Research Hospital, Chengdu, 610072 China

**Keywords:** Recurrent spontaneous abortion, Icariin, Treg cells, Immune tolerance, mTOR signaling pathway

## Abstract

Recurrent spontaneous abortion (RSA) is the most common pregnancy-related complication, affecting 1–5% of pregnancies. Currently, immune imbalance at the maternal–fetal interface is one of the main causes of recurrent abortion. Icariin (ICA) can exert immunomodulatory effects in a variety of autoimmune diseases. Nevertheless, it has not been reported for use in recurrent abortion. In this study, to clarify the effects and mechanisms of ICA for recurrent abortion, female mice CBA/J were randomly divided into Normal group, RSA group and RSA + ICA group. From 0.5 days of pregnancy to 12.5 days, the RSA + ICA group was subjected to orally ICA (50 mg/Kg) daily, and the Normal group and the RSA group were given with an equal volume of distilled water. The results showed the amount of reabsorbed embryo in the RSA group was significantly higher than that in the normal-pregnancy group. However, ICA treatment showed a rescue effect on spontaneous abortion in RSA mice. ICA was able to increase the ratio of the labyrinth to total placental area in abortion-prone model. Further investigation showed that ICA treatment can expand the regulatory T cell (Treg) population in mice prone to abortion, significantly decrease the populations of Th1 cells, and reduce the expression of pro-inflammatory factors. Additionally, ICA treatment was able to decrease the expression of mechanical target of rapamycin (mTOR) in the placenta. ICA may increase Treg cell expansion and reducing pro-inflammatory factors expression via the mTOR pathway, then reducing placental inflammation and improving pregnancy outcomes in abortion-prone mice.

## Introduction

Recurrent spontaneous abortion (RSA) is a serious complication during pregnancy. 1–5% of reproductive-age women are affected. Women over 30 years old are at the highest risk. It is generally defined as three or more consecutive pregnancies that occur before the 24th week of gestation [1]. The aetiology of RSA includes either genetic, structural, infective, endocrine, immune, or unexplained causes. Thrombotic disease is also considered to be a cause of recurrent pregnancy abortion. Unfortunately, the cause of RSA is still unknown in 60% of the cases, and that group is defined as unexplained RSA [2]. However, frequent inspection of these unexplained RSA cases has revealed a potential immunologic alteration associated with failure of immune tolerance at the maternal–fetal interface [3]. Pregnancy is the growth of a semi-allogeneic fetus in the mother’s uterus, similar to an organ transplant, which can trigger an immune response during pregnancy without being rejected by the maternal immune system [4]. At present, the mechanism by which the fetus can develop in the mother is not clear. Among them, special features of the fetal trophoblast in contact with maternal tissue have been suggested to aid immune tolerance through mechanisms such as low tryptophan levels, lack of accurate activation of NK cells expressed via HLA-G/C, high progesterone levels, lack of classical HLA class I and class II trophoblast expression, and anti-idiotypic network regulation [5, 6]. However, none of these proposed mechanisms can fully explain maternal tolerance to the fetus during pregnancy. A recent study has shown that the immune balance between immune cells at the maternal–fetal interface and their secreted cytokines generates a tolerance microenvironment that plays an important role during pregnancy [7].

Th1 cells, Th17 cells, and Treg cells are mostly differentiated from naive CD4^+^ T cells and play key roles in various autoimmune disease models. Th1 and Th17 are a subset of inflammatory Th lymphocytes that secrete potent pro-inflammatory cytokines to induce inflammatory responses. Many studies have indicated that an abnormal increase of Th1 cells and the imbalance of the Th1/Th2 ratio are important factors causing recurrent miscarriage [8]. Th17 plays a role in the acute rejection of early allografts. Early evidence suggests that excessive Th17 cell counts and high levels of IL-17 and IL-1β have been identified in recurrent spontaneous abortion [9]. Regulatory T (Treg) cells are a unique subgroup of T cells and have immunomodulatory functions. Treg cells can regulate the balance of T cells and inhibit activated CD4^+^ T cells and CD8^+^ T cells, which are connected to a variety of immune diseases [10]. Forkhead box protein 3 (FOXP3) is a specific marker of Treg cells that are expressed in the cytoplasm. It plays an important role in regulating the developmental and functional effects of Treg cells[5]. Treg cells have received more attention in maternal–fetal immune tolerance, and some reports have shown that they are the major participant in this unique environment [11, 12]. Several functional studies have shown that unexplained infertility, preeclampsia and miscarriage are often linked to the deficit in Treg cells’ numbers and functions [13–15]. Additionally, murine abortion can be prevented by transferring Treg cells from normal pregnant mice [16]. Treg cells play a major role in maintaining tolerance to paternal allogeneic antigens, which is a critical step for the success of a pregnancy.

Mechanical target of rapamycin (mTOR), a serine/threonine kinase, is a crucial regulator of cell growth, proliferation, differentiation, and survival. The mTOR pathway integrates multiple environmental and intracellular signals from growth factors, nutrients, oxygen, and energy status to regulate cell survival, growth, proliferation, protein synthesis, autophagy, and metabolism [17]. mTOR involves two functionally and biochemically distinct signalling complexes. mTOR complex 1 (mTORC1) and mTOR complex 2 (mTORC2) belong to the phosphoinositide 3-kinase (PI3K)-related kinase family and regulate two major downstream substrates. p70 ribosomal S6 kinase (p70S6K) and Eif-4E (4E-BP1) can be activated via phosphorylation of the mTORC1 complex, which is the vital translational regulator that promotes cell growth and proliferation [18]. A recent study has shown that mTOR has an important role in the immune response and is a key regulator of immune function. Additionally, studies have reported that mTOR plays a role in regulating a variety of immune cells including neutrophils, macrophages, DCs, NK cells, T cells, and B cells [19, 20]. For example, mTOR affects the maturation of professional antigen-presenting cells, such as DCs. It also plays a vital role in the activation of conventional T cells and the proliferation and function of Treg cells [21]. In addition, the immunosuppressive effect of the mTOR inhibitor rapamycin is beneficial to patients with systemic autoimmune diseases, such as systemic lupus erythematosus (SLE), rheumatoid arthritis, and organ specific autoimmune diseases [22]. Rapamycin may establish long-term immune tolerance in experimental models by inhibiting the differentiation and function of effector T cells and expanding the Treg cell population [23]. Inhibition of mTOR can induce immunosuppression in autoimmune diseases and organ transplantation. Pregnancy is like organ transplantation. Therefore, the study of mTOR inhibition has become a target for the treatment of abortion.

In recent years, Unexplained miscarriages are associated with rejection of the fetus by the maternal immune system leading to the use of immunosuppressive drugs such as lipid emulsions, glucocorticoids, intravenous immunoglobulins, or paternal lymphomonocytes [24]. However, because of the limited results of the study population, there is almost no evidence to prove their efficacy. The relevant immunomodulatory effects have not been elucidated. Icariin (ICA) is a natural prenylated flavonol glycoside that has a potential to treat inflammatory and immunological diseases [25]. In experimental autoimmune encephalomyelitis (EAE), Th1 and Th17 cells were reduced in the spleen and lymph nodes of mice after ICA treatment [26]. In an ovalbumin-induced asthma model mice, the suppression of IL-17 and the increase of *Foxp3* mRNA expression in splenic CD4^+^ T cells have been through ICA treatment, indicating that ICA regulates the Th17/Treg balance [27]. ICA can result in a significant inhibition of IL-17 as well as a notable increase in *Foxp3* mRNA expression, which suggests ICA regulate the imbalance of Treg/Th17 and play a therapeutic effect on SLE [28]. ICA may alleviate osteoarthritis by reducing autophagy in chondrocytes by inhibiting the PI3K/AKT/mTOR signalling pathway [29]. Growing applications and emerging evidence suggest that ICA is a promising natural herbal compound with diverse immune regulating functions. Presently, the CBA/J × DBA/2 J mating combination is an important model for RSA because of constant and relatively high abortion rate, coupled with other inherent advantages of inbred mice such as high experimental repeatability and high inter-individual homogeneity. It has been widely used to study the mechanisms that cause and prevent partner-specific recurrent miscarriage (RM) and preeclampsia (PE). This study applied this model to explore the therapeutic effect and mechanism of ICA on RSA.

### Materials and Methods

#### Mice

Inbred female CBA/J (H-2Kk), 7–8 weeks, were purchased from Huafukang Biological Technology Co. Ltd (Beijing, China). Male DBA/2 J (H-2Kd) and male BALB/c (H-2Kd), 7–8 weeks, were purchased from Slack Laboratory Animal Co. Ltd (Shanghai, China). Mice were kept in the Laboratory Animal Facility at a normal 12-h light/dark cycle and conditioned for 1 week before the start of the experiment. Animal care and experimental procedures were conducted according to the guidelines of the Chinese Animal Care Committee. Male DBA/2 J were mated with female CBA/J to generate the abortion-prone model. Male BALB/c were mated with female CBA/J to generate the normal pregnancy model as previously described.

### Animal Treatments and Groups

Female CBA/J mice were bred with male DBA/2 J or male BALB/c mice (1:1 mate). After overnight cohabitation, a female CBA/J with a vaginal plug designated as day 0.5 of pregnancy. CBA/J females were naturally mated to BALB/c males to create the NP model (*n* = 12) (CBA/J × BALB/c). Mice mated with DBA/2 J mice (CBA/J × DBA/2 J) were randomized and divided into the following groups: (1) RSA group (*n* = 12) and (2) RSA + ICA group (*n* = 12, ICA purchased from Saen Chemical Technology Co, Ltd. Shanghai, China). CBA/J female mice in RSA + ICA group were treated with ICA at a dose of 50 mg/kg/day (dissolved in distilled water, 0.2 ml/mouse/day) daily starting on day 0.5 of gestation until day 12.5. Treatment is done by gavage. The normal group and RSA group were administered the same volume of distilled water. The dose of ICA was chosen based on other mouse model studies and our pre-experiments [30–34].

### Embryo Resorption

On day 12.5 of gestation, the female mice were euthanized and the uteri were removed. The fetal resorption sites were identified by their small size and the appearance of avascular necrosis compared with normal embryos. The resorption rate was calculated by the following formula: R (%) = [Re/(Re + F)] × 100%, where Re is the number of resorbed embryos and F is the number of viable embryos. The sera and the placental were collected for subsequent experiments.

### HE Staining

The placental tissue was fixed in 10% formalin for 48 h, followed by xylene and paraffin embedding. A microtome was used to cut the wax block into 4-μm-thick slices from the midsagittal plane at 45 °C and iron them. Then, dewaxing was conducted as follows: xylene I for 20 min, xylene II for 20 min, 100% ethanol I for 5 min, 100% ethanol II for 5 min, 75% ethanol for 5 min, and rinsed with tap water. Sections were stained with haematoxylin solution for 3–5 min and then rinsed with tap water. They were then treated with haematoxylin differentiation solution and rinsed with tap water. Sections were treated with haematoxylin Scott tap bluing and then rinsed with tap water. Then, they were washed with 85% ethanol for 5 min and then 95% ethanol for 5 min. Finally, sections were stained with eosin dye for 5 min. They were dehydrated as follows: 100% ethanol I for 5 min, 100% ethanol II for 5 min, 100% ethanol III for 5 min, xylene I for 5 min, xylene II for 5 min and then sealed with neutral gum. Stained sections were examined under a microscope (NIKON ECLIPSE E100). Cell Sens Dimension 1.6 software (Olympus, Tokyo, Japan) was used to interpret the stained representative areas. The ratio of labyrinth was calculated as follows: The ratio of labyrinth = the area of labyrinth/the area of the whole placenta.

### Enzyme-Linked Immunosorbent Assay

The mouse blood collected from the orbital vein was left at room temperature for 2 h. It was centrifuged at 3000 g for 20 min, and the supernatant was collected. The level of IL-17A and IFN-γ were measured using commercial ELISA kits (Multi sciences Ltd.) according to the manufacturer’s protocol. In short, the first antibody was diluted and incubated with the collected serum at 37 ℃ for 1 h. After washing several times, add the secondary antibody and incubate it at 37 ℃ for 1 h, then add horseradish peroxidase (HRP) solution and incubate it for another hour. Add the chromogenic substrate 3,3′,5,5′-tetramethyl b-benzidine (TMB) and incubate in the dark for 15 min to stop the reaction quickly. All incubations were performed at room temperature. The absorbance in each microplate was measured at 450 nm using a microplate reader (RT-6100, Rayto Life and Analytical Sciences, China) within 5 min of the reaction.

### Isolation of Placenta-Infiltrating Lymphocytes

The placenta-infiltrating lymphocytes were isolated by the Percoll density-gradient centrifugation. All placentas of each pregnant female mouse were removed from intact embryonic tissue and cut to 1 mm^3^ piece with ophthalmic scissors. Then use 1 mL syringe plunger to grind the tissue and pass in the 40-μm cell filter, the tissue fragments were washed with PBS to obtain tissue cell suspension. After centrifugation, the supernatant was discarded and the pellet was resuspended in 5 mL of 40% Percoll (GE Healthcare Biosciences Piscataway, NJ, USA). Three milliliters of 70% Percoll was slowly added to the cell suspension to create a concentration gradient. The sample was divided into three layers, and white blood cells are obtained between two of the layers [35].

### Flow Cytometry

Lymphocyte types in the placenta were detected by FCM (BD Biosciences, San Jose, CA, USA). The detached cells were washed twice with PBS and resuspended in PBS. Then, the cells were mixed with anti-mouse CD3 (APC) and CD4 (FITC) (Biolegend; San Diego, CA, USA) and incubated for 10 min in the dark. Afterwards, cells were washed in 1 mL of PBS and fixed in 1 mL of fixative for 30 min. Then, they were collected and washed in 1 mL of PBS and incubated in 1 mL of permeabilization reagent in the dark for 30 min. Next, resuspended cells in PBS were incubated with anti-mouse IFN-γ (APC), anti-mouse tumour necrosis factor (TNF) (PE) and anti-mouse FOXP3 (APC) (Biolegend; San Diego, CA, USA). They were incubated in the dark for 10 min, and then, the cells were washed again and incubated in PBS. FCM was conducted to analyze the labelled cells, and FCM data were ed using Flow Jo (BD Biosciences).

### RT-qPCR

The whole placental and decidual unit were isolated independently from the respective embryos, and the implantation site was used as the source of total RNA. The frozen sample was homogenized into a fine powder using liquid nitrogen in a pre-cooled mortar and pestle. Then, the Trizol method was used to extract total RNA from the tissue, and the reverse transcription reaction was used to synthesise cDNA according to the instructions from the reverse transcription kit. Finally, the qPCR reaction was prepared (10μL): 1.0μL template cDNA, 5μL fluorescence quantitative reaction premix, 0.4μL Primer1, 0.4μL Primer2 and 3.2μL ddH_2_O. The reaction conditions were as follows: 95 °C pre-denaturation 3 min → 95 °C denaturation 3 s → 60 °C annealing, extension 32 s, amplification for 40 cycles. 18S ribosomal RNA was used as an internal reference, and the relative expression was quantified using the 2^−ΔΔCt^ method. The normalized results were used for statistical analysis. The PCR primers for TNF were as follows: forward, 5′-AGCCAGGAGGGAGAACAGAAAC-3′ and reverse, 5′-GCCACAAGCAGGAATGAGAAGAG-3′. The PCR primers for IL-1β were as follows: forward, 5′-ATCTCGCAGCAGCACATCAAC-3′ and reverse, 5′-TAGAGCGTCGTCGTGTAGTTG-3′; 18S ribosomal RNA (rRNA) mouse (130-bp product), forward, 5′-TTGACGGAAGGGCACCACCAG-3′ and reverse, 5′-GCACCACCACCCACGGAATCG-3′.

### Western Blot

After collection, mouse placentas were lysed with a radioimmunoprecipitation assay buffer to extract total protein, and the protein concentration was determined using the bicinchoninic acid method (Servicebio, China). The protein solution was added to a 5* reduced protein loading buffer (Servicebio, China), denatured in a boiling water bath for 15 min and stored at − 20 °C. Proteins were separated by SDS-PAGE using a 10% gel and transferred to PVDF membranes (Bio-Rad, USA). Membranes were blocked with 5% non-fat dry milk and incubated with primary antibodies against mTOR (2983S), P-mTOR (Ser2448, 5536S), p70S6K (2708P), P-p70S6K (Thr389, 9234S), Erk (Erk1/2, 9102S), p-ERK (Thr202/Tyr204, 4370S), IL-1β(#ab9722), TNF-α (#3707), and GAPDH overnight at 4 °C. All antibodies were purchased from Cell Signalling Technologies. The protein was then incubated with a secondary antibody for 1 h at 37 °C and visualized using enhance chemiluminescence (Novland, China) detection system. GAPDH as an endogenous control was used to normalize input protein. Band intensities were quantified using ImageJ software.

### Statistical Analysis

Statistical analysis of experimental data was conducted using the Graph Pad Prism software program (version 7.0, GraphPad Software, San Diego, CA). A student’s *t*-test was used to compare the data between the two groups. Three independent experiments were analyzed using a one-way analysis of variance and Tukey’s post-hoc test for statistical analysis. All data are presented as the mean ± Standard error of mean (SEM), and *p* < 0.05 indicates statistical significance.

## Results

### ICA Treatment Increased Fetal Survival in a Spontaneous Abortion Mouse Model

**Fig. 1:**
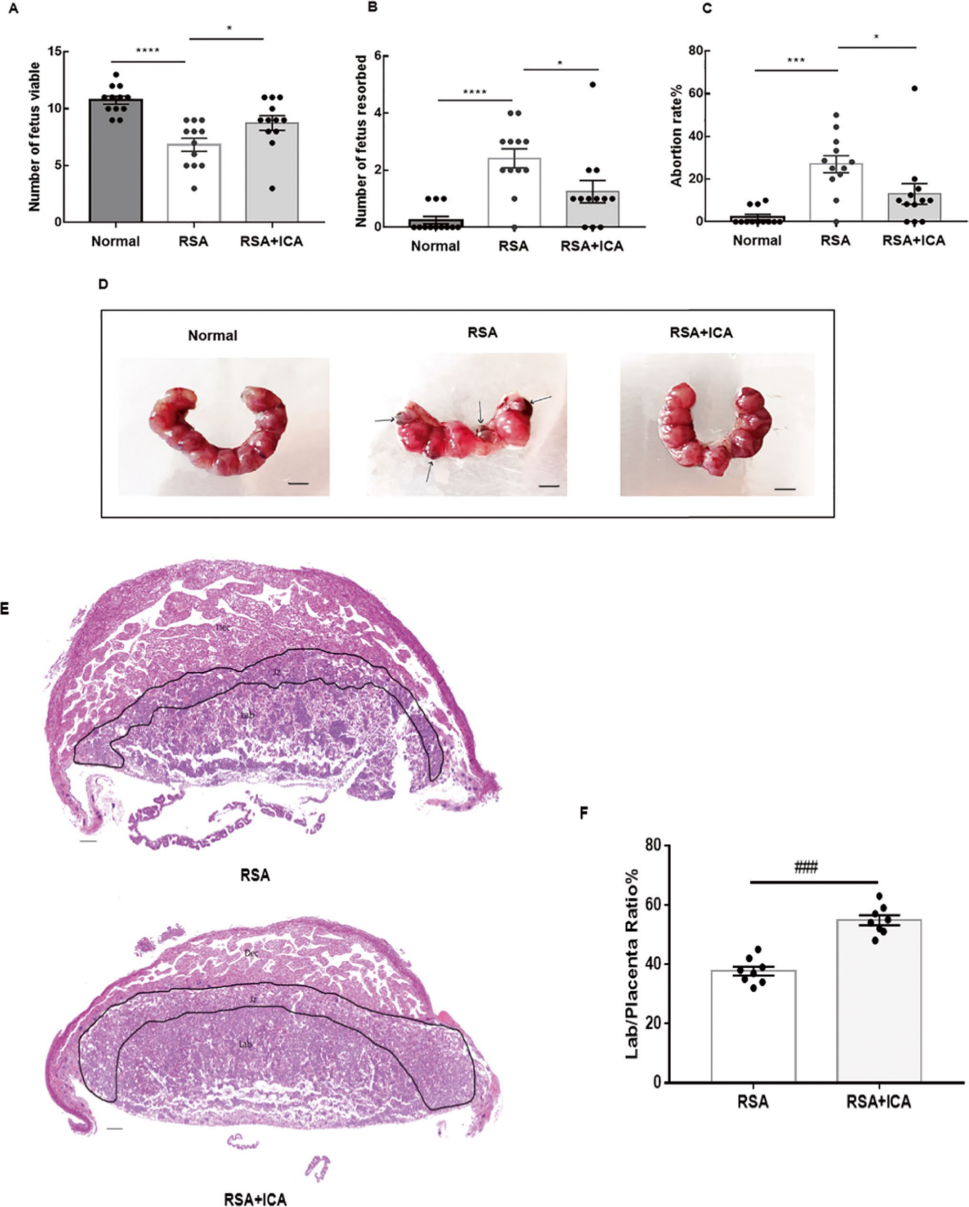
ICA decreased the abortion rate and increased the area of the placental labyrinth in the spontaneous abortion model. **A**–**C** The number of fetus viable, the number of fetus resorbed and the abortion rate in the Normal group, the RSA group and the RSA + ICA group were analyzed separately, and the abortion rate in three group were 2.21 ± 3.86%, 27.00 ± 13.17%, and 13.50 ± 16.03% from left to right (*n* = 12). **D** Representative pictures of Embryo abortion sites of pregnant mice on day 12.5 of pregnancy in each group. The normal group, 0 embryos resorbed; the RSA group, 4 embryos lost; ICA-treated group, 0 embryo resorbed. Black arrows indicate resorbed embryos. Scale bar = 5 mm. **E** H&E staining of midsagittal sections of the placenta in the RSA group and the RSA + ICA group. The placenta is divided into three parts by the black dotted line: the labyrinth, the junction zone, and the decidual. Scale bar = 500 μm. **F** The ratio of labyrinth/fetoplacental area in the RSA group versus the RSA + ICA group, and the percent of labyrinth to fetoplacental area in two group were 37.25 ± 4.96%(RSA), 55.52 ± 2.69% (RSA + ICA) (*n* = 8). *p < 0.05, ###*p* < 0.001, compared to the RSA group, ***p < 0.001, ****p < 0.0001, compared to the normal group. The data in the graph are expressed as the mean ± SEM. Dec: Decidual, Jz: Junction zone, Lab: Labyrinthine

In this study, the number of fetus viable, the number of fetus resorbed, and the fetal absorption rate at 12.5 days of pregnancy in three group were analyzed separately (Fig. 1A-1C). The average abortion rate in Normal group was 2.21% and 27.00% in RSA group. Compared with the Normal group, the embryo resorption rate in the RSA model group was significantly higher (*p* < 0.001). However, the RSA + ICA group had an abortion rate of 13.50%. It was significantly reduced with ICA treatment compared to RSA group (*p* < 0.05). The aborted embryos were a dark red color and small (Fig. 1D), whereas the normal embryos were pink and had a normal shape. These results suggested that ICA treatment showed a rescue effect on spontaneous abortion in abortion-prone mice.

### ICA Treatment Increased the Area Ratio of the Labyrinth to the Placenta in RSA Mice

In a normal pregnancy, the placental labyrinth performs a crucial function in gas exchange and provides nutritional support to the fetus. Developmental defects in the placenta are an important cause of miscarriage [36]. To explore the effect of ICA on the placenta, histopathological changes were identified in different parts of the placenta. The placenta is divided into the decidua (top), the junction zone (middle), and the labyrinth (bottom) (Fig. 1E). The labyrinth zone is outlined by a black dotted line in the figure. Using morphological analysis to show the ratio of the labyrinth to the whole placenta (labyrinth/placenta ratio), the RSA + ICA group had an increased labyrinth area relative to the whole fetoplacental area (*p* < 0.001) compared with the RSA group (Fig. 1F).

### ICA Treatment Has an Anti-inflammatory Effect on the Uteri of Abortion-Prone Mice

**Fig. 2:**
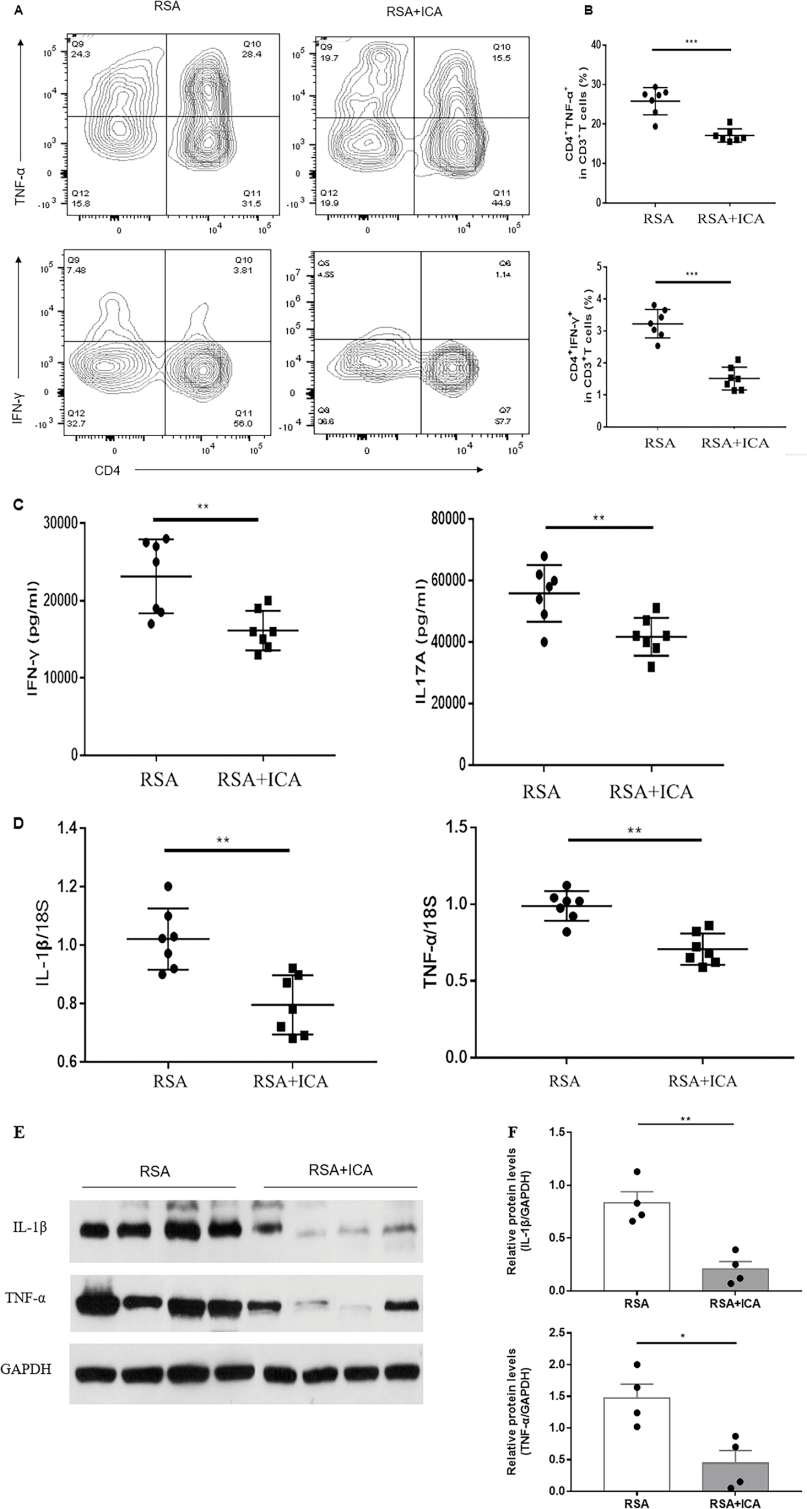
ICA reduced the population of pro-inflammatory cells in the placenta and prevented pro-inflammatory cytokine production. CBA/J female mice were sacrificed at 12.5 days of gestation and the cell suspension was prepared from isolated placental tissues for analysis. **A** Representative CD4^+^IFN-γ^+^ and CD4^+^TNF-α^+^ flow cytometry plots from the placenta of the control group and ICA group are shown. **B** Quantitative analysis of the percentage of CD4^+^IFN-γ^+^ and CD4^+^TNF-α^+^ gated in CD3 + T cell in each group (*n* = 7). **C** Concentrations of pro-inflammatory factors IFN-γ and IL-17A in the serum from each mouse were determined by ELISA (*n* = 7). **D** The transcription of IL-1β and TNF-α to 18S in the abortion-prone and ICA-treated mice were examined using RT-PCR (*n* = 7). **E** The protein expression levels of IL-1β and TNF-α were detected in RSA and ICA-treated mice by western blot analysis. **F** Densitometer analysis results for IL-1β and TNF-α (*n* = 4). *p < 0.05, **p < 0.01, ***p < 0.001, compared to the RSA group. The data in the graph are expressed as the mean ± SEM

Flow cytometry was conducted to analyze lymphocyte populations in the placenta. When the placenta has matured and the maternal–fetal immune regulation becomes active, the placental lymphocytes cells are clearly distributed at 12.5 days of gestation. To examine the phenotype of pro-inflammatory cells, the expressions of CD4^+^IFN-γ^+^ and CD4^+^TNF-α^+^ double-positive cells were examined via flow cytometry. Compared with the RSA group, the population of these cells in the RSA + ICA group was significantly lower (*p* < 0.001) (Figs. 2A and B). The RT-PCR analysis indicated that ICA was able to significantly reduce the transcription of pro-inflammatory factors IL-1β and TNF-α in the placenta (*p* < 0.01) (Fig. 2D). Additionally, the expression of pro-inflammatory cytokines in the placenta was examined according to the ELISA analysis and Western blot. The ELISA showed that the expression of pro-inflammatory factors IFN-γ and IL-17A was significantly lower in the RSA + ICA group (*p* < 0.01) (Fig. 2C) and the expression of IL-1β (*p* < 0.01) and TNF-α (*p* < 0.05) was inhibited after ICA treatment by western blotting (Figs. 2E and F). These results indicate that ICA may regulate the maternal–fetal immune tolerance environment by reducing the number of pro-inflammatory cells and suppressing the expression of pro-inflammatory factors in the placenta.

### ICA Treatment Increased the Population of the Treg-Positive Cell in Abortion-Prone CBA/J × DBA/2 J Mating

**Fig. 3:**
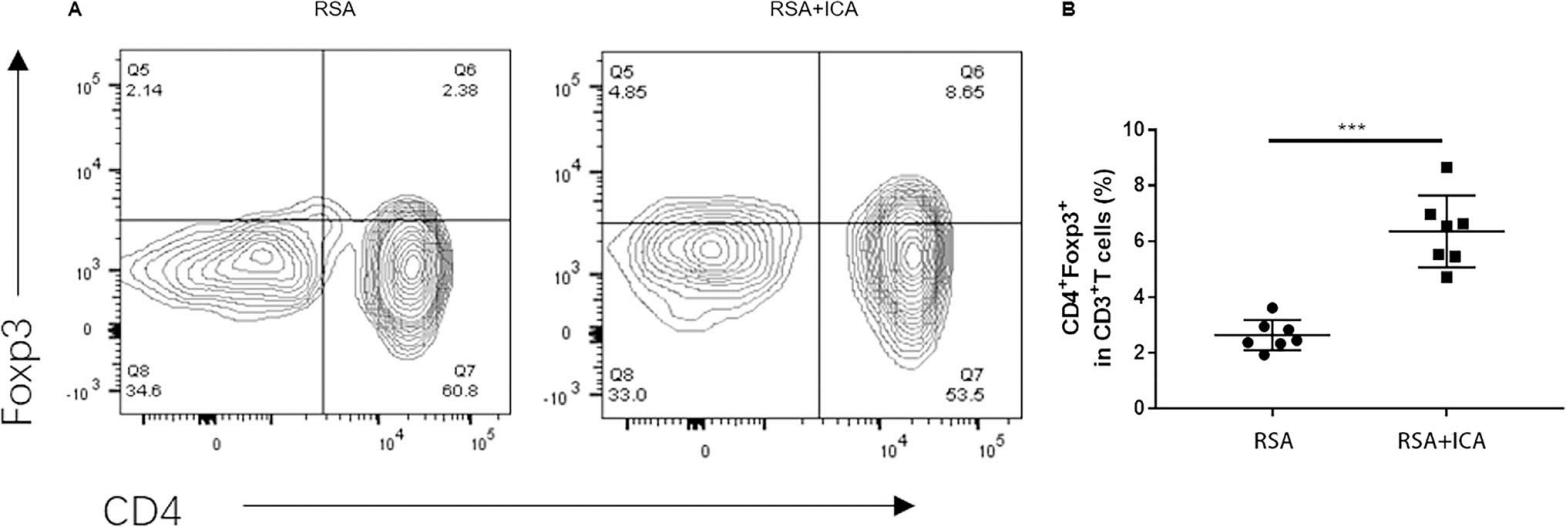
Treg cells increased in the placenta with ICA treatment. The percentage of Treg cells in the placenta was detected by flow cytometry in RSA before and after ICA therapy. **A** Representative CD4^+^Foxp3^+^ in CD3^+^ T cells flow cytometry plots from the placenta of the RSA group and RSA + ICA group are shown. **B** Quantitative analysis of the percentage of CD4^+^Foxp3^+^ T cell cells in each group (*n* = 7). ***p < 0.001, compared to the RSA group. The data in the graph are expressed as the mean ± SEM

To further elucidate the mechanism by which ICA assists female mice to avoid abortion, placental Treg populations were assessed via flow cytometry in pregnant CBA/J mice. Figure 3 shows that the percentage of CD4^+^Foxp3^+^T cell population in RSA + ICA group was significantly increased compared with the RSA group (*p* < 0.001). This finding suggests that ICA can reduce the rate of miscarriage in mice by increasing the number of Treg cells.

### ICA Treatment Affects the Abortion Model via the mTOR Pathway

**Fig. 4:**
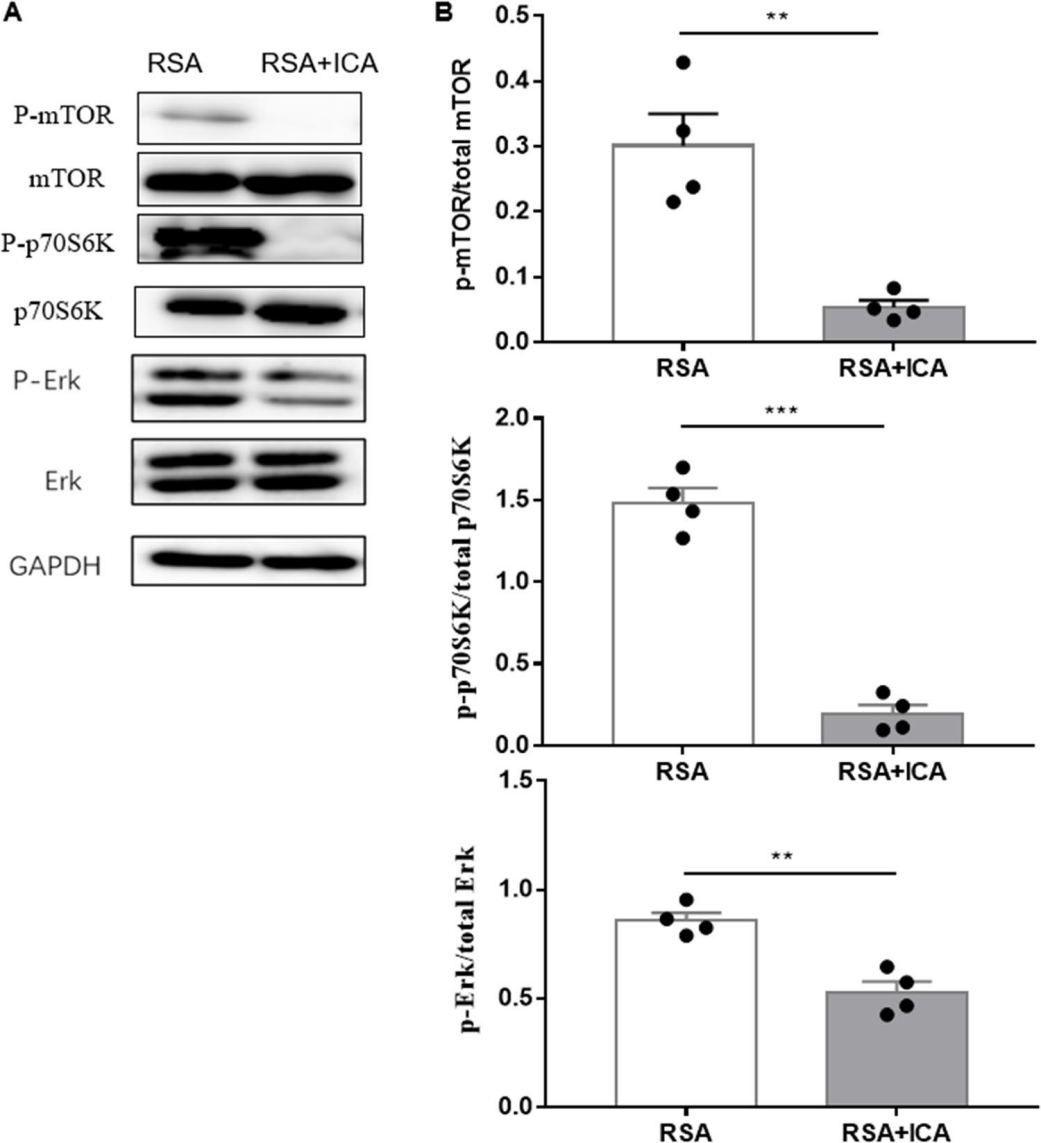
ICA inhibits the Erk/mTOR/P70S6K signaling pathway. **A** Expression levels of mTOR, p-mTOR, Erk, p-Erk, p70S6K, and p-p70S6K proteins were determined by Western blot analysis. **B** Quantitative data of p-mTOR, p-p70S6K p-Erk phospho/total proteins (*n* = 4). **P < 0.01, ***P < 0.001, compared to the RSA group. The data in the graph are expressed as the mean ± SEM. mTOR, mammalian target of rapamycin; p70S6K, phosphoprotein 70 ribosomal protein S6 kinase, Erk, extracellular regulated protein kinases, p-, phosphorylation

To determine whether the mTOR pathway is related to the decreased abortion rate in the abortion mouse model after ICA treatment, the activity of mTOR and upstream and downstream proteins was analyzed via western blots. The results showed that ICA treatment exhibited a significant inhibitory effect on mTOR signalling (Fig. 4). The relative protein expression level of P-mTOR (*p* < 0.01), P-p70S6K (*p* < 0.001), and P-Erk (*p* < 0.01) in the placenta was significantly reduced in the RSA + ICA group compared with the RSA group. This finding supports the involvement of the mTOR pathway with ICA treatment.

## Discussion

RSA is a common disease among women during their childbearing age, which seriously affects women’s physical and mental health. The incidence of recurrent miscarriage is gradually increasing [1]. The cause of repeated miscarriages might be related to the maternal immune system attacking the fetus [37]. Therefore, it is thought that induction of maternal immunosuppression can improve abortion outcome. There is no specific treatment for it. Thus, new drugs with great efficiency and low toxicity will be highly beneficial to RSA patients. ICA is a natural prenylated flavonol glycoside extracted from the plant Epimedium, which is a traditional Chinese medicine with anti-inflammatory and immunomodulatory effects [38]. At present, icariin has been studied in pregnancy model, and it has been reported that icariin has protective effect on mouse embryo development [34, 39]. In this study, we explored the role of ICA in a mouse model of spontaneous abortion using FCM and RT-PCR as well as histopathological analysis of pathological change.

As early as 1986, Clark DA conceived of the CBA/J × DBA/2 J mating combination as a spontaneous abortion model in which immune factors played an important role [40]. In the present study, the protective effects of ICA were investigated in a spontaneous abortion murine model in vivo. ICA was administered at 50 mg/kg to CBA/J × DBA/2 J mice from 0.5 to 12.5 days of gestation. To assess the effect of ICA on the abortion model, abortion rates were examined. The result shows that ICA can reduce the miscarriage rate of mice, and Fig. 1 A shows that the average abortion rate in the RSA group is between 20% and 30%, which is consistent with the results of the literature describing the rate of miscarriage [41]. Moreover, at 12.5 days of gestation, there were differences in the appearance and development of embryos in the model group and the abortion group. In normal embryo tissue, the embryos were pink and the fetuses developed normally. By contrast, aborted embryos show avascular necrosis and abnormal fetal development. Therefore, the placenta is an important site to verify the therapeutic effect of ICA. Related placental HE shows that ICA can increase the area ratio of the placental functional area labyrinth in abortion-prone model. All these manifestations indicate that ICA treatment can effectively treat a recurrent miscarriage.

The occurrence of abortion involves the interaction of various types of immune cells and secreted cytokines between mother and fetus, such as the balance between circulating and placental Th1/ Treg /Th17 subsets [42]. Th1 cells mainly produce proinflammatory cytokines, such as tumor necrosis factor-α, IFN-γ, and IL-2, which can stimulate cellular inflammation and participate in the process of fetal rejection [43]. Th17 cells induce protective immunity against extracellular microbes during pregnancy. However, excessive Th17 immunization may induce uncontrolled neutrophil infiltration at the maternal–fetal interface [9]. In our study, we examined the proinflammatory cell Th1 and the proinflammatory factors TNF-α, IFN-γ, IL-17A, and IL-1β. Among them, we used PCR to detect the transcription of TNF-α and IL-1β, but this could not represent the quantification of protein, and there were certain limitations, so we used Western blot to supplement and prove the quantitative reduction of TNF-α and IL-1β protein. Therefore, the number of pro-inflammatory cells was increased and the expression of proinflammatory cytokines was high in the abortion model, which was consistent with previous descriptions. However, these indexes were downregulated after ICA treatment. It can be proved that ICA can alleviate placental inflammation. CD4^+^Foxp3^+^T regulatory cells are the key cells to inhibit autoimmunity and maintain autoimmunity tolerance [44]. The high proportion of these cells is beneficial for maintaining immune tolerance and improving pregnancy outcomes. In this study, flow cytometry was used to detect the expression of Foxp3 in placental tissue lymphocytes, and the results showed that the Foxp3^+^ cells in the ICA-treated mice were significantly higher than in the control group. Therefore, ICA treatment can increase the number of Treg cells and play an immunomodulatory function. This is consistent with the role of ICA in other autoimmune diseases such as EAE and SLE. At present, ICA has been shown to modulate the changes of immune components in the placenta in our study. However, further studies are needed to understand the detailed mechanisms by which icariin improves the outcome of recurrent abortion.

mTOR is a protein kinase that controls cell growth, proliferation and survival. To further explore the possible mechanism of the ICA immune regulation in RSA, we linked recurrent abortion to mTOR. mTOR and related proteins in placenta were detected by Western blot. Extracellular signal-regulated kinase (Erk) is a special type of mitogen-activated protein kinase. There are many cross-talk mechanisms and patterns between Erk and mTOR. For example, the Ras-Erk pathway can cross-activate PI3K-mTORC1 signalling by regulating the expression of PI3K, TSC2, and mTORC1. Additionally, mTOR phosphorylates and activates p70S6K and 4E-BP1, which in turn phosphorylates S6 and triggers the ribosomal 40S subunit to participate in protein translation [45]. mTOR/S6K axis positively regulates Th17 differentiation and negatively regulates Treg differentiation [46]. In the present study, ICA treatment suppressed pathogenic Th17 cells and tended to increase the percentage of protective Tregs. Western blotting also confirmed that the expressions of P-mTOR, P-Erk, and P-p70S6k were decreased in the ICA treatment group, and these changes suggested that ICA may be transduced by inhibiting mTOR-related signalling pathways.

In summary, ICA improves pregnancy outcomes in animal models prone to miscarriage. Interestingly, ICA expands the population of Treg cells and the secretion of pro-inflammatory factors. Its role is played through the mTOR pathway. However, there are still some limitations in this study. Firstly, the indicators detected are not complete. In addition to IFN-γ and TNF-α, there are many other Th1 and Th17 related proteins, such as IL-4, IL-23, and RORγt. Secondly, the efficacy of the different administration methods was not compared. In the next step of our study, we will expand the sample size, improve the detection indicators, and explore the best drug delivery method. In addition, concentration gradients should be designed to further improve the experimental protocol and explore the optimal therapeutic concentration. The in vitro cell model was used to further elucidate the mechanism of ICA in the treatment of RSA.

## Data Availability

Data will be provided on request.
